# Virological Traits of the SARS-CoV-2 BA.2.87.1 Lineage

**DOI:** 10.3390/vaccines12050487

**Published:** 2024-05-01

**Authors:** Lu Zhang, Alexandra Dopfer-Jablonka, Inga Nehlmeier, Amy Kempf, Luise Graichen, Noemí Calderón Hampel, Anne Cossmann, Metodi V. Stankov, Gema Morillas Ramos, Sebastian R. Schulz, Hans-Martin Jäck, Georg M. N. Behrens, Stefan Pöhlmann, Markus Hoffmann

**Affiliations:** 1 Infection Biology Unit, German Primate Center—Leibniz Institute for Primate Research, 37077 Göttingen, Germany; luzhang@dpz.eu (L.Z.); inehlmeier@dpz.eu (I.N.); akempf@dpz.eu (A.K.); lgraichen@dpz.eu (L.G.); spoehlmann@dpz.eu (S.P.); 2 Faculty of Biology and Psychology, Georg-August-University Göttingen, 37073 Göttingen, Germany; 3 Department of Rheumatology and Immunology, Hannover Medical School, 30625 Hannover, Germany; jablonka.alexandra@mh-hannover.de (A.D.-J.); calderonhampel.noemi@mh-hannover.de (N.C.H.); cossmann.anne@mh-hannover.de (A.C.); stankov.metodi@mh-hannover.de (M.V.S.); morillasramos.gema@mh-hannover.de (G.M.R.); behrens.georg@mh-hannover.de (G.M.N.B.); 4 German Center for Infection Research (DZIF), Partner Site Hannover-Braunschweig, 30625 Hannover, Germany; 5 Division of Molecular Immunology, Department of Internal Medicine 3, Friedrich-Alexander University of Erlangen-Nürnberg, 91054 Erlangen, Germanyhans-martin.jaeck@fau.de (H.-M.J.); 6 Center for Individualized Infection Medicine (CiiM), 30625 Hannover, Germany

**Keywords:** SARS-CoV-2, BA.2.87.1, spike protein, host cell entry, ACE2 binding, antibody evasion

## Abstract

Transmissibility and immune evasion of the recently emerged, highly mutated SARS-CoV-2 BA.2.87.1 are unknown. Here, we report that BA.2.87.1 efficiently enters human cells but is more sensitive to antibody-mediated neutralization than the currently dominating JN.1 variant. Acquisition of adaptive mutations might thus be needed for efficient spread in the population.

## 1. Introduction

The emergence and rapid global dominance of the highly mutated Omicron variant in 2021 and its sublineage JN.1 (a derivative of BA.2.86) in 2023 revealed that novel, antigenically distinct variants can rapidly reshape the now fading COVID-19 pandemic. COVID-19 vaccines, including those that are based on mRNA-technology [1,2,3,4,5], have high effectiveness in protecting vulnerable populations (such as elderly and/or immunocompromised individuals) from the development of severe disease or death following SARS-CoV-2 infection [6,7]. While first generation COVID-19 vaccines were based on SARS-CoV-2 lineages that circulated at the beginning of the pandemic, adapted COVID-19 vaccines have been developed to boost immune responses against recently emerged, highly-mutated SARS-CoV-2 lineages, such as BA.1, BA.5, or XBB.1.5 [8,9,10]. However, despite the availability of these safe and immunogenic vaccines, novel SARS-CoV-2 lineages keep emerging and continue to circulate in populations with high vaccination coverage. 

At the end of 2023, a novel SARS-CoV-2 lineage, BA.2.87.1, was detected in eight patients in South Africa and one traveler entering the USA. The BA.2.87.1 lineage harbors 65 mutations in the spike (S) protein (relative to the virus that circulated in Wuhan in early 2020 (Supplementary Figure S1a,b), which facilitates viral entry into cells and constitutes the key target for neutralizing antibodies [11]. Of these 65 mutations, 33 reside within the N-terminal domain (NTD; C15Δ, V16Δ, N17Δ, L18Δ, L19Δ, T20Δ, R21Δ, T22Δ, Q23Δ, L24Δ, P25Δ, P26Δ, A27S, H69Δ, V70Δ, G75D, S98F, V126A, C136Δ, N137Δ, D138Δ, P139Δ, F140Δ, L141Δ, G142Δ, V143Δ, Y144Δ, Y145Δ, H146Δ, W152L, R190S, V213G, and D215G), a region of the spike protein that contributes to cellular binding of SARS-CoV-2, through interaction with different attachment factors such as AXL (tyrosine-protein kinase receptor UFO) or glycans [12,13,14], and is targeted by neutralizing antibodies [15,16,17]. In addition, 19 mutations are located in the receptor binding domain (RBD; G339D, S371F, S373P, S375F, T376A, D405N, R408S, K417T, N440K, K444N, V445G, L452M, N460K, S477N, N481K, E484A, Q498R, N501Y, and Y505H), which engages the cellular receptor ACE2 [18,19] and represents the key target for neutralizing antibodies [20]. Finally, the remaining part of the S1 subunit (residues 1–685) harbors six mutations (D614G, P621S, V642G, H655Y, N679R, and P681H), while the S2 subunit (residues 686–1273), which includes the domains required for membrane fusion and S protein incorporation into the viral membrane [11], contains another seven mutations (S691P, N764K, T791I, D796H, D936G, Q954H, N969K). 

However, it is unknown whether these mutations are compatible with robust entry into human cells and allow for efficient antibody evasion. We addressed these questions using pseudovirus particles (_pp_) bearing the SARS-CoV-2 S protein, which adequately model key aspects of SARS-CoV-2 entry into host cells and antibody-mediated neutralization [21]. Besides particles bearing BA.2.87.1 S (BA.2.87.1_pp_), we included particles pseudotyped with the S proteins of the B.1 lineage (B.1_pp_), which circulated early in the pandemic, the XBB.1.5 lineage (XBB.1.5_pp_), which served as the target lineage for adaptation of the latest COVID-19 mRNA vaccines [10], and the currently prevailing JN.1 lineage (JN.1_pp_). 

## 2. Materials and Methods

### 2.1. Cell Culture

The following cell lines were incubated at 37 °C in a humidified atmosphere containing 5% CO_2_. Vero (African green monkey kidney, female, kidney; CRL-1586, ATCC; RRID:CVCL 0574, kindly provided by Andrea Maisner), 293T (human, female, kidney; ACC-635, DSMZ; RRID:CVCL 0063), Vero cells stably expressing TMPRSS2 (Vero-TMPRSS2; JCRB1819, CellBank Australia (Westmead, Australia); RRID:CVCL_YQ49, kindly provided by Stuart G. Turville) and Huh-7 cells (human, male, liver; JCRB, JCRB0403; RRID: CVCL_0336, kindly provided by Thomas Pietschmann) were cultured using Dulbecco’s modified Eagle medium (DMEM, PAN-Biotech; Aidenbach, Germany), supplemented with 10% fetal bovine serum (FCS, Biochrom; Berlin, Germany), and 1% penicillin (final concentration 100 U/mL) streptomycin (final concentration 0.1 mg/mL of) solution (P/S, PAN-Biotech; Aidenbach, Germany). LoVo cells (human, male, colon; ACC-350, DSMZ [Deutsche Sammlung von Mikroorganismen und Zellkulturen; Braunschweig, Germany]; RRID:CVCL_0399) were cultured using Roswell Park Memorial Institute medium (RPMI, PAN-Biotech; Aidenbach, Germany), supplemented with 10% FCS and 1% P/S solution, whereas Calu-3 cells (human, male, lung; HTB-55, ATCC [American Type Culture Collection; Manassas, VA, USA]; RRID:CVCL_0609, kindly provided by Stephan Ludwig) were cultured using DMEM/F-12 medium (Thermo Fisher Scientific; Waltham, MA, USA), supplemented with 10% FCS, 1% P/S solution, 1x non-essential amino acid solution (from 100x stock, PAN-Biotech; Aidenbach, Germany) and 1 mM sodium pyruvate (PAN-Biotech; Aidenbach, Germany). Calu-3 cells stably expressing the beta-galactosidase omega fragment (Calu-3-Omega) were generated by retroviral transduction and selection with puromycin (Invivogen; San Diego, CA, USA). Calu-3-Omega cells were further maintained in the same medium as parental Calu-3 cells supplemented with 2 µg/mL of puromycin. All cell lines were regularly tested for the absence of mycoplasma contamination and validated by STR analysis, partial sequencing of the cytochrome c oxidase gene, microscopic examination, and/or according to their growth characteristics. Transfection of 293T cells was performed by calcium phosphate precipitation, while BHK-21 cells were transfected using Lipofectamine 2000 (Thermo Fisher Scientific; Waltham, MA, USA) according to the manufacturers’ instructions. 

### 2.2. Expression Plasmids and Sequence Analysis

The expression plasmids pCAGGS-DsRed, pCAGGS-VSV-G, pCG1-sol-ACE2-Fc, pQCXIP_human-ACE2-cMYC, pQCXIP_raccoon dog-ACE2-cMYC, pQCXIP_pangolin-ACE2-cMYC, pQCXIP_mink-ACE2-cMYC pQCXIP_cat-ACE2-cMYC, pQCXIP_mouse-ACE2-cMYC, pQCXIP_Rhinolophus affinis-ACE2-cMYC, pQCXIP_Rhinolophus sinicus-ACE2-cMYC, pCG1-SARS-CoV-2 B.1 SΔ18 (codon-optimized, C-terminal truncation of 18 amino acid residues, GISAID Accession ID: EPI_ISL_425259), pCG1-SARS-CoV-2 XBB.1.5 SΔ18 (codon-optimized, C-terminal truncation of 18 amino acid residues, GISAID Accession ID: EPI_ISL_16239158), pQCXIP-beta-galactosidase alpha fragment and pQCXIP-beta-galactosidase omega fragment have been described before [22,23,24], while the expression plasmid for SARS-CoV-2 JN.1 SΔ18 (GISAID Accession ID: EPI_ISL_18530042) was generated by introduction of mutation L455S into plasmid pCG1-SARS-CoV-2 BA.2.86 SΔ18 (codon-optimized, C-terminal truncation of 18 amino acid residues, GISAID Accession ID: EPI_ISL_18114953) via overlap-extension PCR and sequence integrity was confirmed by Sanger sequence using a commercial service (Microsynth SeqLab; Göttingen, Germany). The pCG1 expression plasmid was a kind gift from Roberto Cattaneo. Information on SARS-CoV-2 lineages and S protein sequences was collected from the GISAID (Global Initiative on Sharing All Influenza Data) (https://gisaid.org/, accessed on 20 February 2024) and CoV-Spectrum (https://cov-spectrum.org/, accessed on 20 February 2024) databases. 

### 2.3. Production of Pseudovirus Particles and Cell Entry Studies

First, 293T cells transfected to express the respective S protein, vesicular stomatitis virus glycoprotein (VSV-G) or DsRed (negative control) were inoculated with VSV-G-transcomplemented VSV*ΔG(FLuc) (kindly provided by Gert Zimmer) [25]. Following an incubation period of 1 h at 37 °C and 5% CO_2_, the supernatant was removed and the cells were washed with phosphate-buffered saline (PBS). Then, medium containing anti-VSV-G antibody (supernatant of I1-hybridoma cells; ATCC no. CRL-2700) was added to all cells except those expressing VSV-G, which instead received medium without antibody. Following an incubation period of 16–18 h, the supernatant was transferred into a sterile centrifuge tube and centrifuged for 10 min at 4000× *g* in order to pellet the cellular debris, before the clarified supernatant was used for experiments or stored at −80 °C until further use. Cell entry studies were conducted with target cells seeded in 96-well plates. For experiments assessing the ability of S proteins to use human or animal ACE2 orthologs as receptors, BHK-21 cells were transfected to express the respective ACE2 ortholog (or no ACE2) prior to infection. In the ACE2-blockade experiments, Vero cells were preincubated (30 min, 37 °C) with different concentrations of anti-ACE2 antibody (10108-MM36, Sino Biologicals; Beijing, China), whereas for experiments addressing the dependency of S protein-driven cell entry on TMPRSS2 and cathepsin L, Vero and Calu-3 cells were preincubated (2 h, 37 °C) with different concentrations of MDL28170 (Santa Cruz; Dallas, TX, USA) or camostat mesylate (Sigma Aldrich; St. Louis, MO, USA) before pseudovirus particles were added. Following the addition of identical volumes of pseudovirus particles, the target cells incubated for 16–18 h at 37 °C and 5% CO_2_ before cell entry efficiency was assessed. For this, the activity of virus-encoded firefly luciferase in the cell lysates was determined. The cells were lysed by incubation (30 min, room temperature) with PBS containing 0.5% Tergitol (Carl Roth; Karlsruhe, Germany). Then, the lysates were transferred to white 96-well plates and luciferase substrate (Beetle-Juice, PJK; Kleinblittersdorf, Germany) was added, before luminescence was quantified using a Hidex Sense plate luminometer (Hidex; Turku Finland).

### 2.4. Analysis of S Protein Processing and Particle Incorporation

Particles bearing the respective S protein (or no S protein, control) were concentrated by high-speed centrifugation (13,300 rpm, 90 min, 4 °C) through a sucrose cushion (20% *w*/*v* sucrose in PBS), before the supernatant was removed and the sucrose cushion was mixed with 1 volume of 2x Sample buffer (0.06 M Tris-HCl, 20% glycerol, 4% SDS, 5% beta-mercaptoethanol, 0.4% bromophenol blue, 2 mM EDTA) and incubated at 96 °C for 15 min. Next, the lysates were subjected to SDS–PAGE and the proteins were blotted onto nitrocellulose membranes (Hartenstein; Würzburg, Germany). The membranes were blocked in PBS-T (PBS with 0.05% Tween 20, Carl-Roth; Karlsruhe, Germany) containing 5% bovine serum albumin (BSA, Carl-Roth; Karlsruhe, Germany) for 30 min, before they were probed with primary antibody overnight at 4 °C. For detection of S proteins, anti-SARS-CoV-2 (2019-nCoV) Spike S2 antibody (rabbit, 1:2000 in PBS-T containing 5% BSA; SIN-40590-T62, Biozol; Eching, Germany;) was used, while the vesicular stomatitis virus matrix protein (VSV-M) was detected as a loading control using an anti-VSV-M [23H12] antibody (mouse, 1:1000 in PBS-T containing 5% skim milk powder; EB0011, Kerafast; Shirley, MA, USA). Next, the membranes were washed with PBS-T and incubated for 1 h at 4 °C with horseradish peroxidase-conjugated secondary antibody (S protein detection: anti-rabbit IgG (H+L)-HRPO; 1:2000 in PBS-T containing 5% skim milk powder; 111-035-003, Dianova; Eching, Germany; VSV-M detection: anti-mouse IgG (H+L)-HRPO; 1:2000 in PBS-T containing 5% skim milk powder; 115-035-045, Dianova; Eching, Germany). Finally, the membranes were washed with PBS-T and protein bands were detected using the ChemoCam imaging system with ChemoStar Professional software version 1.54d (Intas Science Imaging Instruments; Göttingen, Germany) and an in-house prepared chemiluminescence solution (0.1 M Tris-HCl [pH 8.6], 250 g/mL luminol, 0.1 mg/mL para-hydroxycoumaric acid, 0.3 percent hydrogen peroxide).

### 2.5. Analysis of S Protein-Driven Cell–Cell Fusion

Effector 293T cells transfected to express the respective S proteins (or empty vector) along with the beta-galactosidase alpha fragment were washed, resuspended in medium, seeded on top of Calu-3-Omega (Calu-3 target cells stably expressing the beta-galactosidase omega fragment) and incubated for 18 h. Next, S protein-driven cell-cell fusion was analyzed. For this, a beta-galactosidase substrate (Gal-Screen, Thermo Fisher Scientific; Waltham, MA, USA) was added, and the cells were incubated for 90 min before the lysates were transferred into white plates and the luminescence was recorded using a Hidex Sense plate luminometer (Hidex; Turku, Finland).

### 2.6. Analysis of S Protein Cell Surface Expression and ACE2 Binding Efficiency

293T cells transfected to express the respective S proteins (or no S protein, control) were washed with PBS, resuspended in PBS-B (PBS with 1% BSA) and pelleted by centrifugation (600× *g*, 5 min, room temperature). Next, the supernatant was aspirated and the cells were resuspended in PBS-B, before the cell suspension was split into two reaction tubes for the detection of S protein cell surface expression and ACE2 binding. (i) S protein cell surface expression: Cells were incubated for 1 h at 4 °C with anti-SARS-CoV-2 S2 subunit antibody (mouse, 1:100 in PBS-B; GTX632604, Biozol; Eching, Germany) in a Rotospin test tube rotator disk (IKA; Staufen, Germany). Thereafter, the cells were pelleted by centrifugation (600× *g*, 5 min, room temperature) and washed with PBS-, before they were incubated for 1 h at 4 °C with Alexa Fluor-488-conjugated anti-mouse antibody (1:200 in PBS-B; A-10667, Thermo Fisher Scientific; Waltham, MA, USA). Next, the cells were pelleted by centrifugation (600× *g*, 5 min, room temperature), washed with PBS-B, fixed with 1% paraformaldehyde solution (30 min, room temperature), washed again and resuspended in PBS-B, before S protein cell surface expression was analyzed using a ID7000 Spectral Cell Analyzer and ID7000 software version 1.1.8.18211 (Sony Biotechnology, San Jose, CA, USA). (ii) ACE2 binding: Cells were incubated for 1 h at 4 °C with soluble human ACE2-Fc (concentrated supernatant of 293T cells transfected with pCG1-sol-ACE2-Fc; 1:20 in PBS-B) in a Rotospin test tube rotator disk (IKA; Staufen, Germany). Thereafter, the cells were pelleted by centrifugation (600× *g*, 5 min, room temperature) and washed with PBS-B, before they were incubated for 1 h at 4 °C with Alexa Fluor-488-conjugated anti-human antibody (1:200 in PBS-B; A-11013, Thermo Fisher Scientific; Waltham, MA, USA). Next, the cells were pelleted by centrifugation (600× *g*, 5 min, room temperature), washed with PBS-B, fixed with 1% paraformaldehyde solution (30 min, room temperature), washed again and resuspended in PBS-B before ACE2 binding was analyzed using a ID7000 Spectral Cell Analyzer and ID7000 software version 1.1.8.18211 (Sony Biotechnology, San Jose, CA, USA). Finally, for each S protein, ACE2 binding was normalized to S protein surface expression.

### 2.7. Ethics Committee Approval and Enrollment of Study Participants

Collection and analysis of plasma samples was performed as part of the COVID-19 Contact (CoCo) Study (German Clinical Trial Registry, DRKS00021152) and have been approved by the Internal Review Board of Hannover Medical School (institutional review board no. 8973_BO-K_2020, last amendment September 2023). All participants provided written informed consent and received no compensation. Of note, the CoCo study is a prospective observational study that monitors anti-SARS-CoV-2 IgG and immune responses in health care professionals at Hannover Medical School and in individuals with potential SARS-CoV-2 contact. 

### 2.8. Plasma Samples 

Cohort 1, vaccinated individuals without a history of SARS-CoV-2 infection who received the XBB.1.5-adapted booster vaccine, which received the recommendation for marketing authorization by the European Medicines Agency (EMA) and approval by the U.S. Food and Drug Administration (FDA) in August/September 2023, as their last vaccination; cohort 2, vaccinated individuals with a history of SARS-CoV-2 infection who received the XBB.1.5-adapted booster vaccine as their last vaccination; cohort 3, vaccinated individuals who did not receive the XBB.1.5-adapted booster vaccine and have a history of one SARS-CoV-2 infection between 11/2023 and 12/2023; cohort 4, vaccinated individuals who did not receive the XBB.1.5-adapted booster vaccine and have a history of two SARS-CoV-2 infections, the last of which occurring between 11/2023 and 12/2023. SARS-CoV-2 S1-specific IgG titers were quantified with the anti-SARS-CoV-2-QuantiVac-ELISA (IgG) (EUROIMMUN; Lübeck, Germany) and the SARS-CoV-2 infection-free status of cohort 1 was confirmed by the absence of anti-SARS-CoV-2 NCP IgG by the anti-SARS-CoV-2 ELISA (NCP) (EUROIMMUN; Lübeck, Germany). Specific information on the plasma samples is summarized in Table 1 and Supplementary Table S1. Of note, none of the participants with a history of SARS-CoV-2 infection developed severe disease that required hospitalization. Before the experiments, the plasma samples were heat-inactivated by incubation at 56 °C for 30 min. 

### 2.9. Neutralization Assay 

Particles bearing the respective S protein were mixed with different concentrations of mAb (range: 0.2 ng/mL to 2 µg/mL) or dilutions of blood plasma (range: 1:25 to 1:6400) and incubated for 30 min at 37 °C, before being inoculated onto Vero cells. Following an incubation period of 16–18 h, their neutralization efficiency was analyzed. For this, entry was normalized to samples without mAb/plasma (set as 0% inhibition). Further, the mAb concentration or plasma dilution leading to half-maximal inhibition (mAb, Effective concentration 50, EC50; plasma, neutralizing titer 50, NT50) were calculated based on a non-linear regression model. Of note, the thresholds for neutralization-positive mAbs and plasma samples were defined as EC50 ≤ 5 µg/mL (2.5 times the highest mAb concentration tested) and NT50 ≥ 6.25 (25% of the lowest plasma dilution tested), respectively.

### 2.10. Quantification and Statistical Analysis

Data were analyzed in Microsoft Excel (part of Microsoft Office Professional Plus, version 2016, Microsoft Corporation) and GraphPad Prism version 8.3.0 (GraphPad Software). Two-tailed Student’s *t*-test with Welch correction, two-way analysis of variance with Dunnetts’ posttest, and Wilcoxon matched-pairs signed rank test were used to analyze statistical significance (the statistical method of the individual experiments is indicated in the figure legends). Only *p* values of 0.05 or lower were considered as statistically significant (ns [not significant], *p* > 0.05; *, *p* ≤ 0.05; **, *p* ≤ 0.01; ***, *p* ≤ 0.001). 

## 3. Results

### 3.1. BA.2.87.1 Efficiently Enters and Fuses Human Cells

**Figure 1:**
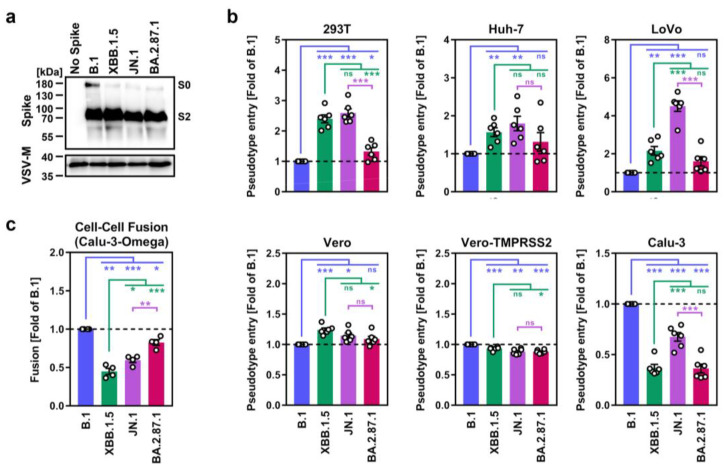
Host cell entry and cell–cell fusion properties of the SARS-CoV-2 BA.2.87.1 lineage. (**a**) Processing and particle incorporation of the BA.2.87.1 S protein. Presented are representative data from a single biological replicate and results were confirmed in five additional biological replicates. (**b**) Entry efficiency of the BA.2.87.1 lineage. Presented are mean data from six biological replicates, conducted with four technical replicates, with cell entry normalized against particles harboring the B.1 S protein (set as 1). Error bars represent the standard error of the mean (SEM). (**c**) Cell–cell fusion capacity of the BA.2.87.1 lineage. Presented are the mean data from four biological replicates, conducted with three technical replicates. Fusion driven by the B.1 S protein was set as 1. Error bars indicate the SEM. Statistical significance was analyzed by two-tailed Students’ *t*-test with Welch correction (*p* > 0.05, not significant [ns]; *p* ≤ 0.05, *; *p* ≤ 0.01, **; *p* ≤ 0.001, ***).

We started our assessment by the production of pseudovirus particles bearing the different S proteins and analyzed S protein incorporation and processing, and S protein driven entry into a panel of cell lines. Immunoblot analysis of pseudovirus particles revealed that all S proteins were efficiently cleaved and incorporated (Figure 1a). Further, all particles efficiently entered a panel of six cell lines (Figure 1b and Supplementary Figure S1c). BA.2.87.1_pp_ entered 293T (human, kidney), Huh-7 (human, liver), LoVo (human, colon) and Vero cells (African green monkey, kidney, ± S protein-priming protease TMPRSS2) with similar efficiency as B.1_pp_ and JN.1_pp_, except for 293T and LoVo cells, which were more susceptible to JN.1_pp_. For Calu-3 cells (human, lung), entry of B.1_pp_ was highest, followed by JN.1_pp_, whereas XBB.1.5_pp_ and BA.2.87.1_pp_ entry was less efficient. 

The ability of the S protein to fuse infected with uninfected cells is believed to contribute to COVID-19 pathogenesis [26,27,28], which is why we assessed the capacity of BA.2.87.1 S to drive cell–cell fusion using a split beta-galactosidase reporter assay (Figure 1c and Supplementary Figure S1d). For this, 293T cells transiently overexpressing the beta-galactosidase alpha-fragment jointly with one of the different S proteins were co-incubated with Calu-3 cells that stably express the beta-galactosidase omega-fragment and cell–cell fusion was quantified by measuring the activity of the reconstituted beta-galactosidase. Using this approach, it was observed that BA.2.87.1 S displayed significantly higher cell–cell fusion capacity compared with XBB.1.5 S and JN.1 S, reaching levels observed for the B.1 S protein. 

### 3.2. BA.2.87.1 Efficiently Utilizes Human and Animal ACE2 as Entry Receptors

**Figure 2:**
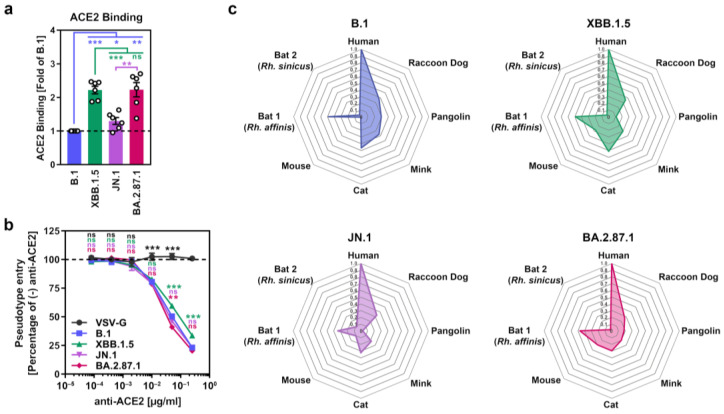
ACE2 usage of the SARS-CoV-2 BA.2.87.1 lineage. (**a**) Soluble human ACE2 binding by the BA.2.87.1 S protein. Presented are mean ACE2 binding data from six biological replicates, conducted with a single technical replicate, and ACE2 binding was corrected for S protein cell surface expression and normalized using the B.1 S protein as the reference (=1). Error bars indicate the SEM. (**b**) Impact of ACE2 blockade on cell entry of the BA.2.87.1 lineage. Presented are mean data from three biological replicates, conducted with four technical replicates, with cell entry in the absence of antibody used as the reference (set as 100%). Error bars represent the SEM. (**c**) Utilization of mammalian ACE2 orthologs by the BA.2.87.1 lineage. Net plots present the mean data from three biological replicates, conducted with four technical replicates, and data were normalized to human ACE2 (set as 1). For panel (**a**) statistical significance was analyzed by two-tailed Students’ *t*-test with Welch correction, while for panel (**b**) statistical significance was analyzed by two-way ANOVA with Dunnett’s posttest (*p* > 0.05, not significant [ns]; *p* ≤ 0.05, *; *p* ≤ 0.01, **; *p* ≤ 0.001, ***).

Next, we analyzed the ability of BA.2.87.1 S to engage the SARS-CoV-2 receptor ACE2. We first assessed S protein binding to soluble human ACE2 (consisting of the ACE2 ectodomain fused to the Fc portion of human immunoglobulin G) by flow cytometry and corrected ACE2 binding of the different S proteins to their respective cell surface expression levels (Supplementary Figure S2b). Using this strategy, we found that BA.2.87.1 S and XBB.1.5 S bound soluble human ACE2 with comparable efficiency while ACE2 binding of B.1 S and JN.1 S was significantly reduced (Figure 2a and Supplementary Figure S2a,b). However, antibody-mediated inhibition of ACE2 engagement did not reveal major differences in ACE2 dependency for Vero cell entry of pseudoviruses bearing the different S proteins (Figure 2b). Moreover, by inoculation of BHK-21 cells transiently overexpressing ACE2 orthologs of different mammalian species with pseudoviruses bearing the different S proteins under study, it was observed that all four S proteins could comparably utilize diverse mammalian ACE2 orthologs as entry receptors, with the exception of pangolin ACE2 (highest for B.1_pp_) and mouse ACE2 (lowest for B.1_pp_) (Figure 2c and Supplementary Figure S2c). Thus, the BA.2.87.1 lineage efficiently binds human ACE2 and robustly enters and fuses human cells, although entry into Calu-3 lung cells is reduced compared to JN.1. 

### 3.3. Lung Cell Entry of BA.2.87.1 Depends on TMPRSS2 

**Figure 3:**
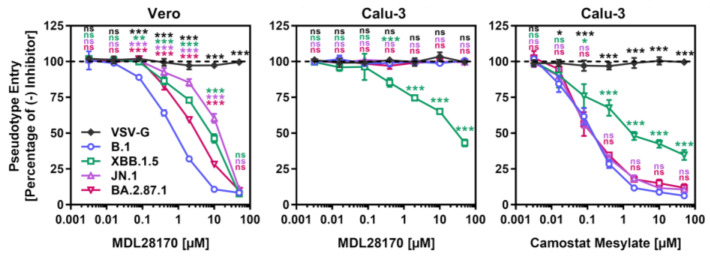
Dependency of the SARS-CoV-2 BA.2.87.1 lineage on TMPRSS2 for lung cell entry. Pseudotype particles harboring the indicated S proteins were inoculated onto Vero and Calu-3 cells that were preincubated with MDL28170 or camostat mesylate and entry was analyzed. Presented are mean data from three biological replicates, conducted with four technical replicates, with cell entry in the absence of inhibitor used as the reference (set as 100%). Error bars represent the SEM. Statistical significance was analyzed by two-way ANOVA with Dunnett’s posttest (*p* > 0.05, not significant [ns]; *p* ≤ 0.05, *; *p* ≤ 0.01, **; *p* ≤ 0.001, ***).

Most Omicron sublineages show a reduced capacity to employ TMPRSS2 for cell entry, which has been linked to diminished lung cell entry and reduced virulence [29,30,31]. Therefore, we evaluated the dependency of BA.2.87.1_pp_ on TMPRSS2 for lung cell entry using the cathepsin L inhibitor MDL28170 and the TMPRSS2 inhibitor camostat mesylate (Figure 3). For this, target cells (Vero and Calu-3) were pre-incubated with different concentrations of inhibitor before pseudoviruses were added. MDL28170 reduced Vero kidney cell entry of all particles analyzed but had no impact on Calu-3 lung cell entry of B.1_pp_, JN.1_pp_ or BA.2.87.1_pp_, while XBB.1.5_pp_ entry into lung Calu-3 cells was diminished. Camostat mesylate inhibited Calu-3 cell entry of all particles, with entry of B.1_pp_, JN.1_pp_ and BA.2.87.1_pp_ being more affected than entry of XBB.1.5_pp_. Finally, neither of the inhibitors reduced entry of control particles bearing the vesicular stomatitis virus glycoprotein (VSV-G). Thus, BA.2.78.1 deviates from the other Omicron sublineage in its ability to efficiently employ TMPRSS2 for lung cell entry.

### 3.4. Few Therapeutic Monoclonal Antibodies Neutralize BA.2.87.1

**Figure 4:**
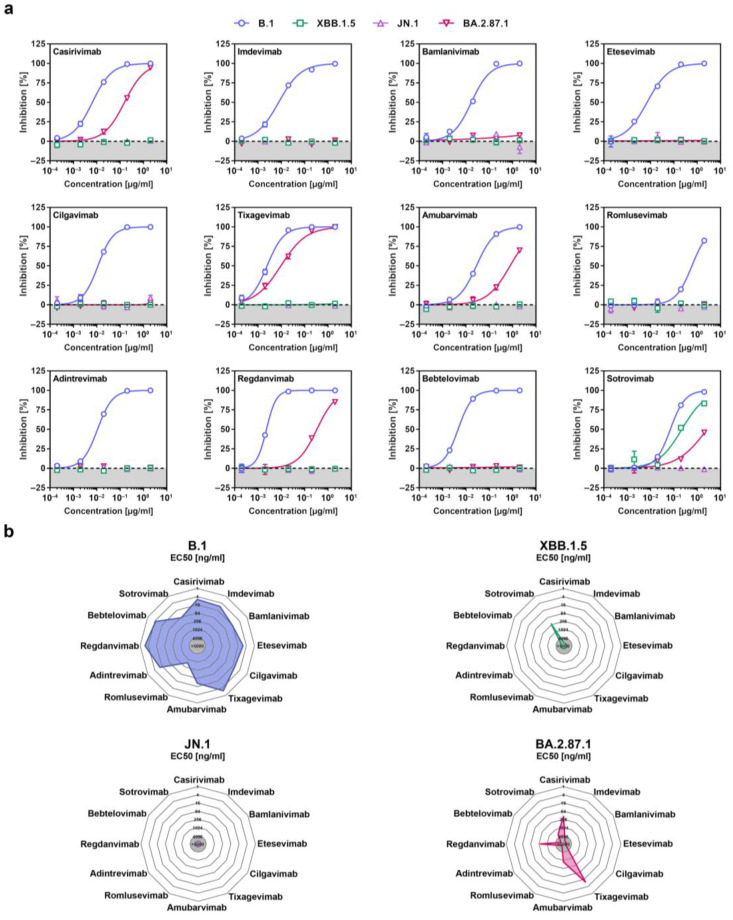
Sensitivity of the SARS-CoV-2 BA.2.87.1 lineage to neutralization by monoclonal antibodies. (**a**) Pseudotype particles harboring the indicated S proteins were incubated with different concentrations of the indicated monoclonal antibodies (mAb) before being inoculated onto Vero cells and cell entry was measured. Presented are the mean data from three biological replicates, conducted with four technical replicates, and cell entry was normalized against entry in the absence of mAb (set as 0% inhibition). (**b**) Net plots indicate the effective dose 50 (EC50) values calculated from the data presented in panel (**a**).

Recombinant monoclonal antibodies (mAb) have been successfully used for COVID-19 therapy but contemporary SARS-CoV-2 lineages developed resistance against most or all of them [32], which is why the FDA suspended or revoked emergency use authorization for several mAbs, including Casirivimab and Imdevimab (marketed as Ronapreve), Bamlanivimab and Etesevimab (marketed as Evusheld), and Sotrovimab (marketed as Xevudy). Pseudovirus particles bearing the different S proteins were pre-incubated with serially diluted mAb before they were added to Vero cells and the reduction in the infectivity of mAb-exposed pseudoviruses to non-mAb-exposed pseudoviruses was used to determine the neutralizing activity of the different mAbs. Using a panel of twelve mAbs that were previously approved for COVID-19 therapy or are currently under development, we found that five of them (Casirivimab, Tixagevimab, Amubarvimab, Regdanvimab and Sotrovimab), displayed neutralizing activity against BA.2.87.1_pp_ and should constitute suitable treatment options (Figure 4a,b and Supplementary Figure S3). In comparison, only Sotrovimab was effective against XBB.1.5_pp_ and none of the mAbs neutralized JN.1_pp_. 

### 3.5. Less Neutralization Evasion by BA.2.87.1 Compared to JN.1

**Figure 5:**
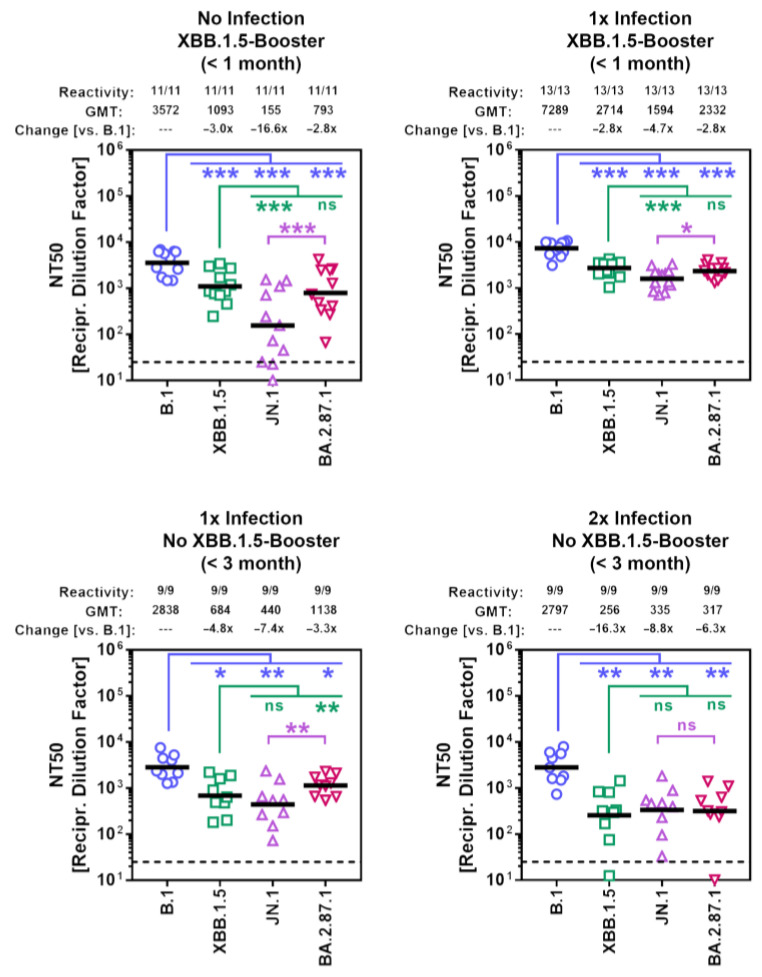
Sensitivity of the BA.2.87.1 lineage to neutralization by antibodies in the blood plasma of individuals with different immunization backgrounds. Pseudotype particles harboring the indicated S proteins were incubated with different dilutions of plasma before being inoculated onto Vero cells. Cell entry was normalized against entry in the absence of plasma (set as 0% inhibition) and the neutralizing titer 50 values were calculated based on a nonlinear regression model. Presented are the geometric mean titers (GMT) from a single biological replicate, conducted with four technical replicates. Information above the graphs include response rates (proportion of plasma samples with neutralizing activity), GMT values, and median fold GMT changes compared to particles bearing the B.1 S protein. Please also see Table 1, Supplementary Table S1 and Figures S4 and S5 for additional information. Statistical significance was assessed by Wilcoxon matched-pairs signed rank test (*p* > 0.05, ns; *p* ≤ 0.05, *; *p* ≤ 0.01, **; *p* ≤ 0.001, ***).

Finally, we studied the sensitivity of BA.2.87.1 to neutralization by antibodies induced upon vaccination or vaccination plus breakthrough infection. Cohorts 1 and 2 included participants who recently received the XBB.1.5-adapted COVID-19 mRNA vaccine from BioNTech (raxtozinameran). Members of cohort 1 had no history of SARS-CoV-2 infection while members of cohort 2 had documented SARS-CoV-2 infection between 01/2022 and 03/2023 (Table 1, Supplementary Table S1 and Figure S4). Cohorts 3 and 4 included participants without XBB.1.5-booster vaccination, who experienced one (cohort 3) or two (cohort 4) SARS-CoV-2 infections with the most recent infection occurring during the JN.1 wave. Of note, all participants received at least four vaccinations with non-XBB.1.5-adapted COVID-19 vaccines and their plasma samples were collected within three months after the last infection or vaccination (Supplementary Table S1 and Supplementary Figure S4). Pseudovirus particles bearing the S proteins under study were pre-incubated with serial blood plasma dilutions before being added to Vero cells and the reduction in pseudovirus infectivity (compared to the infectivity of pseudoviruses that were not exposed to blood plasma) was used to determine the neutralizing activity of the plasma samples. For all four cohorts, the highest neutralizing activity was measured for B.1_pp_ (geometric mean titer = 2797–7289), while neutralization of JN.1_pp_ was the lowest (~5–7-fold reduction compared to B.1_pp_) with the exception of cohort 4 (Figure 5 and Supplementary Figure S5). Importantly, although BA.2.87.1_pp_ displayed substantial resistance to antibody-mediated neutralization independent of the cohort analyzed, neutralization evasion was less efficient compared to JN.1_pp_, with the exception of cohort 4.

## 4. Discussion

Our initial virological assessment of the BA.2.87.1 lineage revealed that it efficiently utilizes human and animal ACE2 orthologs as receptors and robustly enters human cell lines. However, cell entry of BA.2.87.1_pp_ was found to be reduced compared to JN.1_pp_. Calu-3 lung cell entry of BA.2.87.1_pp_ was highly dependent on the activity of the cellular serine protease TMPRSS2, a trait that is shared with lineages dominating the pre-Omicron era and the recently emerged BA.2.86 lineage [32,33]. With respect to antibody-mediated neutralization, we found that BA.2.87.1 can be neutralized by Casirivimab, Tixagevimab, Amubarvimab, Regdanvimab and Sotrovimab, which could constitute suitable treatment options in case of BA.2.78.1 spread. In addition, BA.2.87.1_pp_ evaded neutralization by antibodies present in the plasma of individuals with diverse immune backgrounds but antibody evasion was reduced compared to JN.1_pp_. 

The following limitations apply to our study. First, pseudovirus particles and cell lines were used to assess BA.2.87.1 host cell entry and its neutralization. Thus, our results await confirmation with authentic SARS-CoV-2 BA.2.87.1 and primary cell cultures and organoids. Second, the pathogenic potential of BA.2.87.1 remains to be analyzed using in vivo models. Third, due to the small sample size for the four cohorts, a detailed analysis on the potential impact of biological factors (e.g., age, gender, or comorbidities) on neutralization efficiency was not possible. Fourth, all plasma samples were collected within three months after the XBB.1.5 booster vaccination or last infection. Thus, it remains to be determine whether differences in neutralization efficiencies for the tested SARS-CoV-2 lineages become more or less pronounced after an extended period of time. Fifth, for cohorts 3 and 4, no specific information on the SARS-CoV-2 lineages that caused infection is available. Sixth, neutralization sensitivity of the SARS-CoV-2 BA.2.87.1 lineage may differ in cohorts with immune backgrounds distinct from the ones examined in the present study. Finally, no plasma samples from individuals without a history of vaccination were tested.

## 5. Conclusions

Based on the data obtained in this study it seems unlikely that BA.2.87.1 will efficiently spread in regions where JN.1 is dominant. However, BA.2.87.1 may still be able to spread in locations where the JN.1 prevalence is low and may acquire additional mutations that improve its transmissibility and/or immune evasion. In sum, this study provides valuable information on the virological traits of the BA.2.87.1 lineage that support political decision makers and medical personnel to determine whether changes in containment and treatment strategies are required.

## Figures and Tables

**Table 1:** General information and immunization background of plasma donors (please see Supplementary Table S1 for details.

Cohort (n)	General Information		Immunization History				Anti-Spike IgG (BAU/mL) *
	Male-to-Female Ratio	Age (Years)	Vaccinations	XBB.1.5 Booster	Infection(s)	Days since Last Immunization	
1 (n = 11)	4/7	range: 25–74; median = 48	range: 5–8; median = 5	Yes	No	range: 15–21; median = 16	range: 1114–6627; median = 2531
2 (n = 13)	6/7	range: 29–62; median = 44	range: 4–5; median = 5	Yes	Yes (n = 1)	range: 15–17; median = 16	range: 1261–6090; median = 2467
3 (n = 9)	3/6	range: 31–64; median = 56	range: 3–4; median = 3	No	Yes (n = 1)	range: 44–88; median = 60	range: 768–5541; median = 2796
4 (n = 9)	2/7	range: 36–58; median = 50	range: 3–4; median = 3	No	Yes (n = 2)	range: 44–81; median = 65	range: 882–5937; median = 1519

* BAU/mL = binding antibody units per mL.

## Data Availability

Raw data are available upon request. This study did not generate code. All materials and reagents will be made available upon installment of a material transfer agreement.

## Supplementary Materials

The following supporting information can be downloaded at: https://www.mdpi.com/article/10.3390/vaccines12050487/s1, Figure S1: Spike protein mutations, host cell entry and cell–cell fusion of the SARS-CoV-2 BA.2.87.1 lineage; Figure S2: ACE2 usage of the SARS-CoV-2 BA.2.87.1 lineage. Figure S3: Overview of BA.2.87.1-specific S protein mutations in the context of epitopes recognized by therapeutic monoclonal antibodies; Figure S4: Immune background of the four cohorts analyzed; Figure S5: Neutralization sensitivity of the SARS-CoV-2 BA.2.87.1 lineage; Table S1: Plasma information.
