# Supplementary material for: Virological Traits of the SARS-CoV-2 BA.2.87.1 Lineage
Source: Vaccines (Basel). 2024 May 1;12(5):487. doi: 10.3390/vaccines12050487 (PMC11125805; doi:10.3390/vaccines12050487)
Supplement: Supplementary file 1 [file vaccines-12-00487-s001.zip › Supplementary figure legends_R2.pdf]

Supplementary figure legends

Supplementary figure S1: Spike protein mutations, host cell entry and cell-cell fusion of the SARS-CoV-2 BA.2.87.1 lineage.

(a) Evolutionary steps leading to JN.1 and BA.2.87.1 (only S protein-specific mutations are shown). **(b)** S protein mutations of B.1, XBB.1.5, BA.2, JN.1 and BA.2.87.1 compared to the Wuhan-Hu-01 isolate. Abbreviations: NTD, N-terminal domain; RBD, receptor-binding domain, pre-S1/S2, region between RBD and S1/S2 cleavage site. **(c)** Cell line tropism and entry efficiency of the SARS-CoV-2 BA.2.87.1 lineage. Particles bearing the indicated S proteins, vesicular stomatitis glycoprotein (VSV-G, positive control), or no viral glycoprotein (negative control) were inoculated onto the indicated cell lines and entry was analyzed at 16–18 h post inoculation by measuring firefly luciferase activity in cell lysates. Presented are the mean data from six biological replicates, conducted with four technical replicates, and cell entry was normalized against the assay background (signals obtained from particles bearing no viral glycoprotein, set as 1). Error bars represent the SEM. **(d)** S protein-driven cell-cell fusion. Effector 293T cells transfected to express the indicated S protein along with the beta-galactosidase alpha fragment were mixed and coincubated for 18h with target Calu-3 cells stably expressing the beta-galactosidase omega fragment. Next, S protein-driven cell-cell fusion was analyzed by quantification of reconstituted beta-galactosidase activity in cell lysates. Presented are the mean data from four biological replicates, conducted with three technical replicates, and fusion was normalized against the assay background (signals obtained after coincubation of target cells with effector cells that did not express S protein, set as 1). Error bars indicate the SEM.

Supplementary figure S2: ACE2 usage of the SARS-CoV-2 BA.2.87.1 lineage.

(a) ACE2 binding efficiency of SARS-CoV-2 S proteins. 293T cells expressing the indicated S proteins following transfection were incubated with soluble human ACE2-Fc and Alexa Fluor-488-conjugated anti-human antibody, before ACE2 binding was analyzed by flow cytometry. Presented are the mean fluorescence intensity (MFI) data from six biological replicates, conducted with a single technical replicate. Error bars indicate the standard deviation (SD). **(b)** Cell surface expression of SARS-CoV-2 S proteins. 293T cells expressing the indicated S proteins following transfection were incubated with anti-SARS-CoV-2 S protein S2 subunit and Alexa Fluor-488-conjugated anti-mouse secondary antibodies, before S protein surface expression was analyzed by flow cytometry. Presented are the mean MFI data from six biological replicates, conducted with a single technical replicate. Error bars indicate the SD. **(c)** Utilization of mammalian ACE2 orthologues by SARS-CoV-2 S proteins. Particles bearing the indicated S proteins or vesicular stomatitis glycoprotein (VSV-G, positive control) were inoculated onto BHK-21 cells expressing the indicated ACE2 orthologues (or no ACE2, control) following transfection and entry efficiency was analyzed at 16–18 h post inoculation by measuring firefly luciferase activity in cell lysates. Presented are the mean data from three biological replicates, conducted with four technical replicates, and changes in cell entry due to ACE2 orthologue expression were calculated using signals obtained from cells expressing no ACE2 as reference (set as 1). Error bars represent the SEM.

Supplementary figure S3: Overview of BA.2.87.1-specific S protein mutations in the context of epitopes recognized by therapeutic monoclonal antibodies.

Summary of RBD-specific mutations (blue) in the context of the XBB.1.5, JN.1 and BA.2.87.1 S proteins (numbering according to the S protein of the SARS-CoV-2 Wuhan-Hu-01 isolate). Residues that directly engage ACE2 are highlighted in yellow, while residues that form epitopes recognized by therapeutic monoclonal antibodies are highlighted in pink.

Supplementary figure S4: Immune background of the four cohorts analyzed.

Cohort 1: Immunization with four to seven doses of non-XBB.1.5-adapted COVID-19 vaccines, followed by a single dose with the XBB.1.5-adapted COVID-19 vaccine of BioNTech/Pfizer. No history of SARS-CoV-2 infection. Cohort 2: Immunization with three to four doses of non-XBB.1.5-adapted COVID-19 vaccines and history of a single SARS-CoV-2 infection between 01/2022 and 03/2023, followed by a single dose with the XBB.1.5-adapted COVID-19 vaccine of BioNTech/Pfizer. Cohort 3: Immunization with three to four doses of non-XBB.1.5-adapted COVID-19 vaccines and history of a single SARS-CoV-2 infection between 11/2023 and 12/2023; no history of vaccination with an XBB.1.5-adapted COVID-19 vaccine. Cohort 4: Immunization with three to four doses of non-XBB.1.5-adapted COVID-19 vaccines and history of two SARS-CoV-2 infections, the last of which happening between 11/2023 and 12/2023; no history of vaccination with an XBB.1.5-adapted COVID-19 vaccine.

Supplementary Figure S5: Neutralization sensitivity of the SARS-CoV-2 BA.2.87.1 lineage.

Individual neutralization data for plasma samples of the four cohorts. Presented are the mean data from one biological replicates, conducted with four technical replicates, and cell entry was normalized against particles incubated in the absence of plasma (set as 0% inhibition). Error bars indicate the SD.
