# Supplementary material for: Virological Traits of the SARS-CoV-2 BA.2.87.1 Lineage
Source: Vaccines (Basel). 2024 May 1;12(5):487. doi: 10.3390/vaccines12050487 (PMC11125805; doi:10.3390/vaccines12050487)
Supplement: Supplementary file 1 [file vaccines-12-00487-s001.zip › Table S1_R2_clean.pdf]

Supplementary Table S1: Information on plasma samples

General information						Vaccination status	Infection status		
**Sample ID**	**Cohort**	**Gender**	**Age (years)**	**Days since last immunization[a]**	**IgG (BAU/ml)[b]**	**Vaccination history**	**Infected (Yes/No)**	**Date of infection(s)**	**Most prevalent lineage(s) at the time of infection**
10170	1	Female	26	15	6627	V#1: Yes (n.i.); V#2: Yes (n.i.); V#3: Yes (n.i.); V#4: BNT (B.1/BA.5); V#5: BNT (XBB.1.5)	No	n.a.	n.a.
10179	1	Female	30	16	5110	V#1: BNT (B.1); V#2: BNT (B.1); V#3: BNT (B.1); V#4: BNT (B.1); V#5: BNT (XBB.1.5)	No	n.a.	n.a.
10182	1	Female	42	15	2125	V#1: BNT (B.1); V#2: BNT (B.1); V#3: BNT (B.1); V#4: BNT (B.1/BA.5); V#5: BNT (XBB.1.5)	No	n.a.	n.a.
10186	1	Male	74	15	1882	V#1: AZD; V#2: AZD; V#3: BNT (B.1); V#4: MOD (B.1); V#5: BNT (XBB.1.5)	No	n.a.	n.a.
10192	1	Female	48	16	3523	V#1: BNT (B.1); V#2: BNT (B.1); V#3: BNT (B.1); V#4: BNT (B.1/BA.5); V#5: BNT (XBB.1.5)	No	n.a.	n.a.
10194	1	Male	61	16	1114	V#1: BNT (B.1); V#2: BNT (B.1); V#3: BNT (B.1); V#4: BNT (B.1); V#5: BNT (XBB.1.5)	No	n.a.	n.a.
10197	1	Female	57	16	2531	V#1: BNT (B.1); V#2: BNT (B.1); V#3: BNT (B.1); V#4: BNT (B.1); V#5: BNT (B.1/BA.5); V#6: BNT (XBB.1.5)	No	n.a.	n.a.
10198	1	Female	64	16	6560	V#1: AZD; V#2: AZD; V#3: BNT (B.1); V#4: BNT (B.1); V#5: BNT (XBB.1.5)	No	n.a.	n.a.

10200	1	Male	57	16	2106	V#1: BNT (B.1); V#2: BNT (B.1); V#3: BNT (B.1); V#4: Yes (n.i.); V#5: Yes (n.i.); V#6: BNT (B.1); V#7: Yes (n.i.); V#8: BNT (XBB.1.5)	No	n.a.	n.a.
10215	1	Male	38	16	2202	V#1: BNT (B.1); V#2: BNT (B.1); V#3: BNT (B.1); V#4: BNT (B.1); V#5: BNT (XBB.1.5)	No	n.a.	n.a.
10223	1	Female	25	21	4461	V#1: AZD; V#2: AZD; V#3: BNT (B.1); V#4: BNT (B.1/BA.5); V#5: BNT (XBB.1.5)	No	n.a.	n.a.
10167	2	Male	44	16	1552	V#1: BNT (B.1); V#2: BNT (B.1); V#3: BNT (B.1); V#4: BNT (XBB.1.5)	Yes	15.04.2022	BA.2
10172	2	Male	50	15	3066	V#1: AZD; V#2: BNT (B.1); V#3: BNT (B.1); V#4: BNT (B.1/BA.5); V#5: BNT (XBB.1.5)	Yes	15.03.2023	XBB.1.5, CH.1.1, XBB.1.9
10178	2	Female	56	15	3434	V#1: BNT (B.1); V#2: BNT (B.1); V#3: BNT (B.1); V#4: BNT (B.1); V#5: BNT (XBB.1.5)	Yes	29.07.2022	BA.5
10180	2	Male	41	16	2797	V#1: AZD; V#2: BNT (B.1); V#3: BNT (B.1); V#4: BNT (B.1/BA.5); V#5: BNT (XBB.1.5)	Yes	14.02.2022	BA.1, BA.2
10185	2	Female	62	16	1690	V#1: BNT (B.1); V#2: BNT (B.1); V#3: BNT (B.1); V#4: BNT (B.1); V#5: BNT (XBB.1.5)	Yes	13.01.2023	BA.5, CH.1.1, XBB.1.5
10188	2	Female	34	16	1952	V#1: BNT (B.1); V#2: BNT (B.1); V#3: BNT (B.1); V#4: BNT (B.1); V#5: BNT (XBB.1.5)	Yes	07.01.2022	BA.1
10189	2	Female	45	16	2467	V#1: AZD; V#2: BNT (B.1); V#3: BNT (B.1); V#4: BNT (B.1); V#5: BNT (XBB.1.5)	Yes	15.07.2022	BA.5
10191	2	Female	29	16	1395	V#1: BNT (B.1); V#2: BNT (B.1); V#3: BNT (B.1); V#4: BNT (XBB.1.5)	Yes	16.02.2022	BA.1, BA.2
10201	2	Male	43	16	5395	V#1: BNT (B.1); V#2: BNT (B.1); V#3: MOD	Yes	15.03.2022	BA.1, BA.2

						(B.1); V#4: BNT (B.1/BA.5); V#5: BNT (XBB.1.5)			
10208	2	Female	42	15	1261	V#1: BNT (B.1); V#2: BNT (B.1); V#3: BNT (B.1); V#4: BNT (B.1); V#5: BNT (XBB.1.5)	Yes	26.10.2022	BA.5
10212	2	Female	31	17	3446	V#1: BNT (B.1); V#2: BNT (B.1); V#3: BNT (B.1); V#4: BNT (B.1); V#5: BNT (XBB.1.5)	Yes	21.05.2022	BA.2
10214	2	Male	58	15	6090	V#1: AZD; V#2: BNT (B.1); V#3: BNT (B.1); V#4: BNT (XBB.1.5)	Yes	15.03.2023	XBB.1.5, CH.1.1, XBB.1.9
10224	2	Male	44	16	1552	V#1: BNT (B.1); V#2: BNT (B.1); V#3: BNT (B.1); V#4: BNT (XBB.1.5)	Yes	13.05.2022	BA.2
10396	3	Female	60	53	3275	V#1: MOD; V#2: Yes (n.i.); V#3: Yes (n.i.)	Yes	17.12.2023	JN.1
10410	3	Male	31	44	2796	V#1: BNT (B.1); V#2: BNT (B.1); V#3: BNT (B.1)	Yes	27.12.2023	JN.1
10445	3	Female	56	79	4973	V#1: AZD; V#2: Yes (n.i.); V#3: Yes (n.i.); V#3: BNT (B.1/BA.5)	Yes	25.11.2023	JN.1, BA.2.86.1
10464	3	Female	58	84	3032	V#1: BNT (B.1); V#2: BNT (B.1); V#3: BNT (B.1)	Yes	20.11.2023	JN.1, BA.2.86.1
10503	3	Female	64	47	1597	V#1: AZD; V#2: BNT (B.1); V#3: BNT (B.1); V#4: BNT (B.1/BA.5)	Yes	28.12.2023	JN.1
10518	3	Female	38	46	768	V#1: AZD; V#2: MOD; V#3: MOD	Yes	29.12.2023	JN.1
10543	3	Male	50	60	1522	V#1: BNT (B.1); V#2: Yes (n.i.); V#3: Yes (n.i.)	Yes	16.12.2023	JN.1
10620	3	Male	58	88	2074	V#1: AZD; V#2: BNT (B.1); V#3: BNT (B.1); V#4: BNT (B.1/BA.5)	Yes	19.11.2023	JN.1, BA.2.86.1
10642	3	Female	56	69	5541	V#1: BNT (B.1); V#2: Yes (n.i.); V#3: Yes (n.i.); V#4: BNT (B.1/BA.5)	Yes	09.12.2023	JN.1
10475	4	Female	58	54	5937	V#1: BNT (B.1); V#2: BNT (B.1); V#3: BNT (B.1)	Yes	12.03.2022<br>20.12.2023	BA.1, BA.2<br>JN.1
10504	4	Female	56	44-74	2226		Yes	xx.08.2022	BA.5, BE.1.1

						V#1: BNT (B.1); V#2: BNT (B.1); V#3: BNT (B.1)		xx.12.2023	JN.1
10505	4	Male	54	44-74	882	V#1: BNT (B.1); V#2: BNT (B.1); V#3: BNT (B.1)	Yes	xx.12.2022	BQ.1.1, BF.7
								xx.12.2023	JN.1
10514	4	Male	49	81	1305	V#1: BNT (B.1); V#2: Yes (n.i.); V#3: Yes (n.i.); V#4: BNT (B.1/BA.5)	Yes	15.05.2022	BA.2
								24.11.2023	JN.1, BA.2.86.1
10533	4	Female	57	78	2647	V#1: AZD; V#2: BNT (B.1); V#3: BNT (B.1)	Yes	13.09.2022	BA.5
								28.11.2023	JN.1, BA.2.86.1
10546	4	Female	42	62	1519	V#1: Yes (n.i.); V#2: Yes (n.i.); V#3: Yes (n.i.)	Yes	xx.04.2022	BA.2
								14.12.2023	JN.1
10562	4	Female	48	45-75	1108	V#1: BNT (B.1); V#2: BNT (B.1); V#3: BNT (B.1)	Yes	15.07.2022	BA.5
								xx.12.2023	JN.1
10600	4	Female	36	65	2118	V#1: BNT (B.1); V#2: Yes (n.i.); V#3: Yes (n.i.); V#4: BNT (B.1/BA.5)	Yes	22.12.2022	BQ.1.1, BF.7
								12.12.2023	JN.1
10653	4	Female	50	47-77	924	V#1: AZD; V#2: BNT (B.1); V#3: BNT (B.1)	Yes	xx.10.2022	BA.5
								xx.12.2023	JN.1

Cohort 1: No Infection[c]/XBB.1.5 booster; Cohort 2: One infection//XBB.1.5 booster; Cohort 3: One infection/no XBB.1.5 booster; Cohort 4: Two infections/no XBB.1.5 booster. [a]: For samples without information on the exact date of infection/vaccination a range is provided; [b]: Anti-SARS-CoV-2 S1 IgG titers were determined against ancestral SARS-CoV-2; [c]: SARS-CoV-2 infection-free status of cohort 1 was confirmed by ELISA (= anti-NCP IgG-negative). Abbreviations: AZD, AZD1222/Vaxzevria; BNT (B.1), BNT162b2/Comirnaty; BNT (B.1/BA.5), Comirnaty Original / Omicron BA.4-5; BNT (XBB.1.5) Comirnaty XBB.1.5; MOD (B.1), Spikevax; BAU/ml, binding antibody units per mL; ID, identifier; IgG, immunoglobulin G; V#, vaccination; n.a., not applicable; n.i., no information available. Of note, no information on the batch numbers for the respective vaccines has been documented.
