# Supplementary figures and images for: Virological Traits of the SARS-CoV-2 BA.2.87.1 Lineage
Source: Vaccines (Basel). 2024 May 1;12(5):487. doi: 10.3390/vaccines12050487 (PMC11125805; doi:10.3390/vaccines12050487)

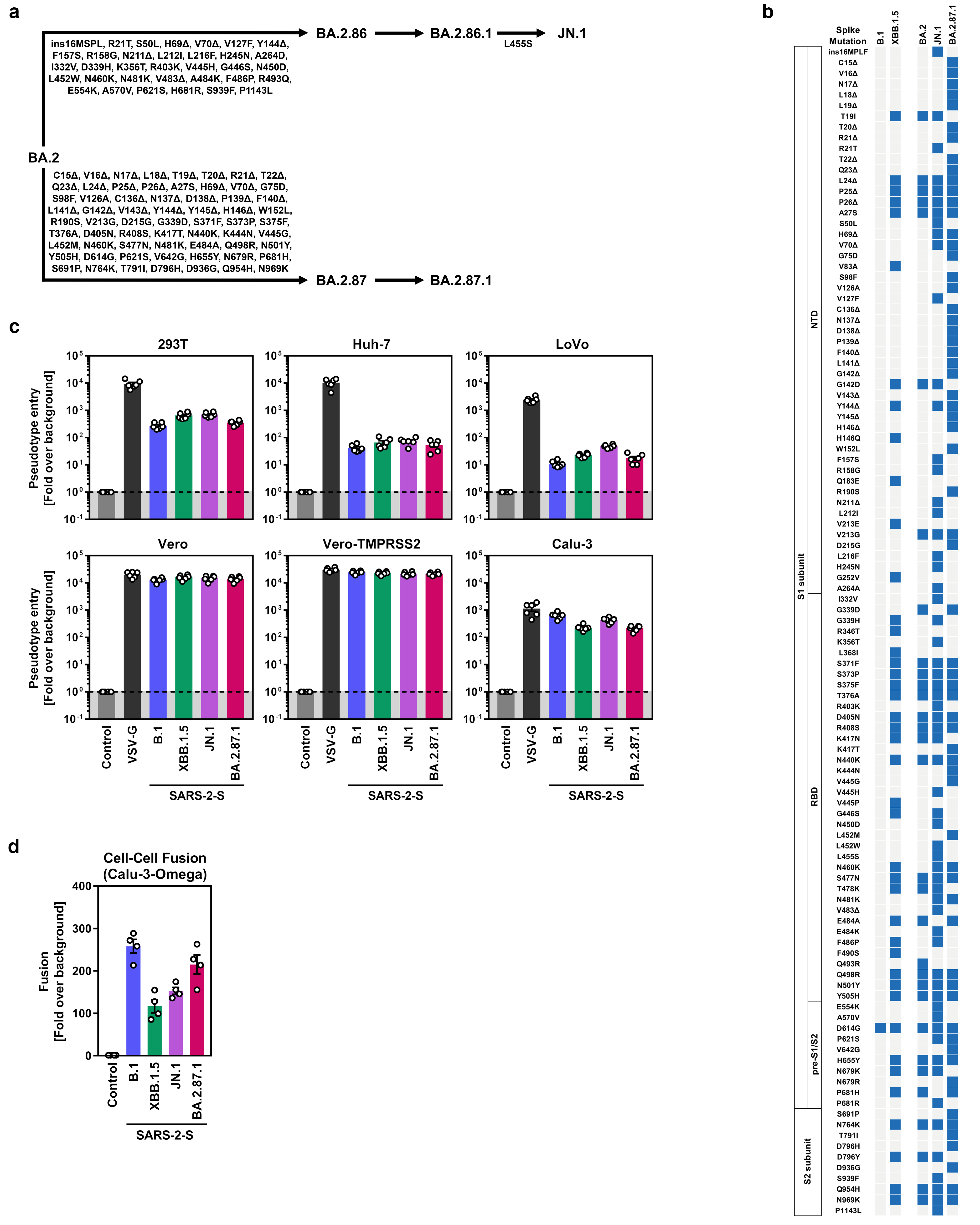

Supplement: Supplementary file 1 [file vaccines-12-00487-s001.zip › SI Figure_1.tif]

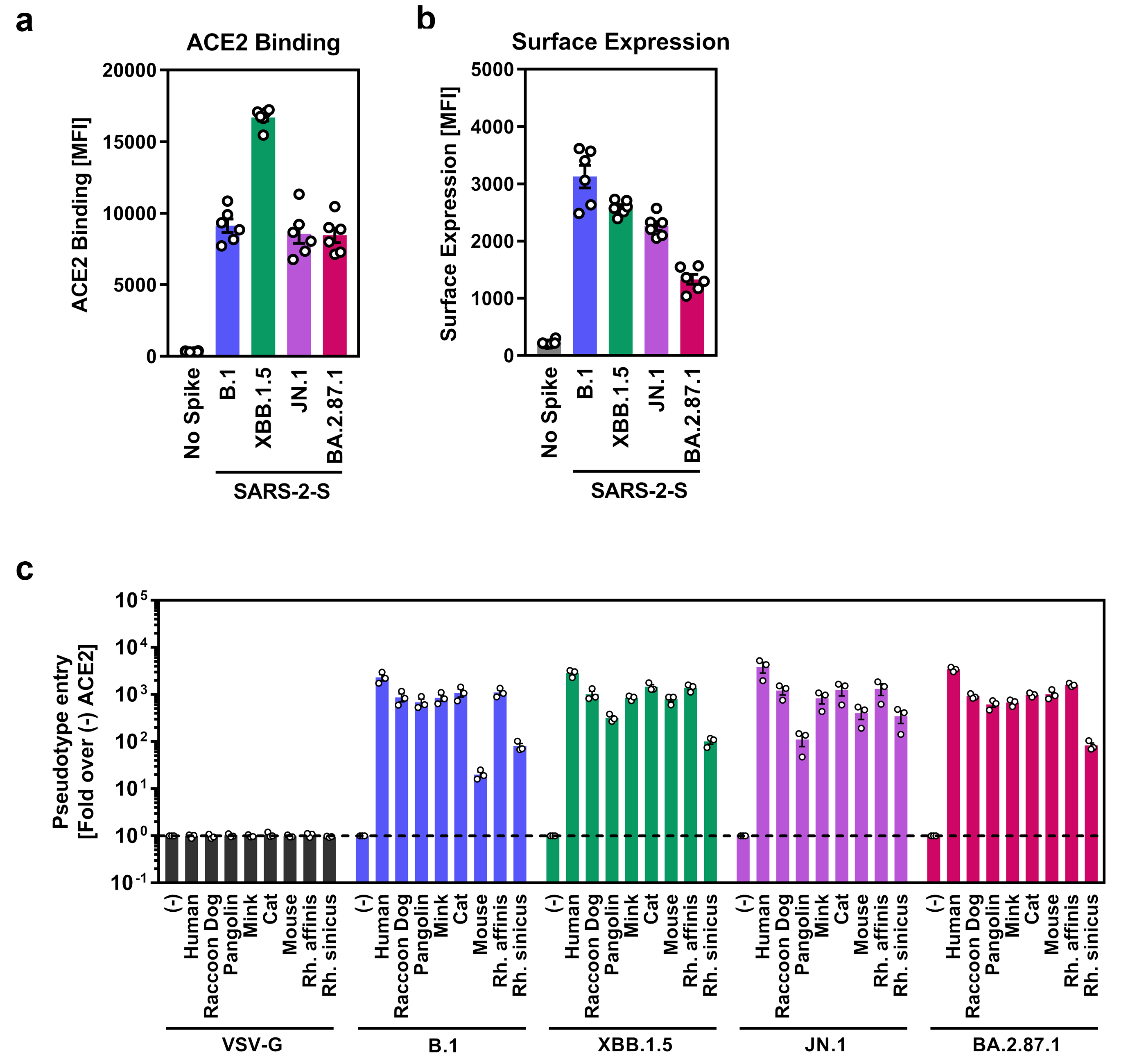

Supplement: Supplementary file 1 [file vaccines-12-00487-s001.zip › SI Figure_2.tif]

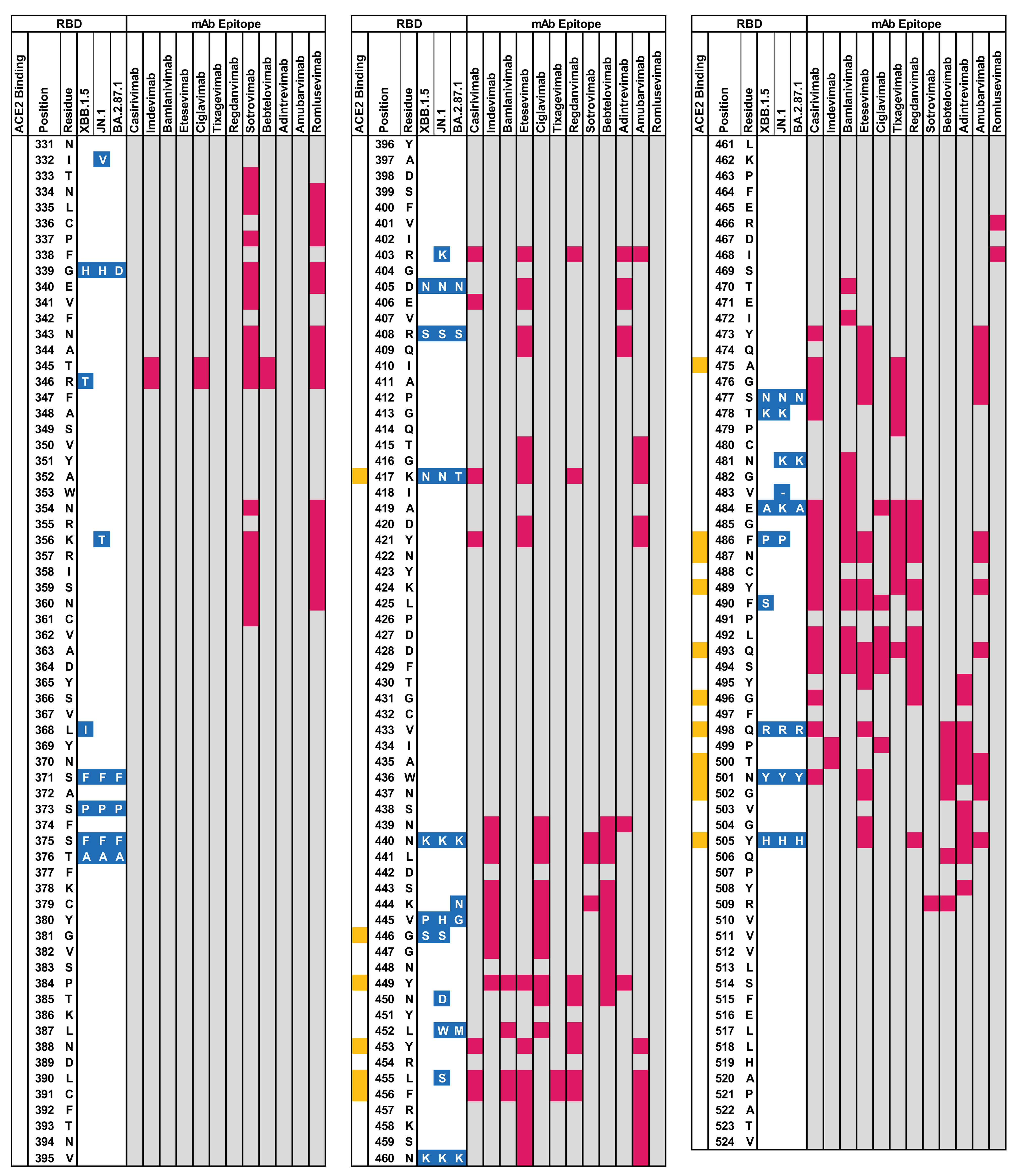

Supplement: Supplementary file 1 [file vaccines-12-00487-s001.zip › SI Figure_3.tif]

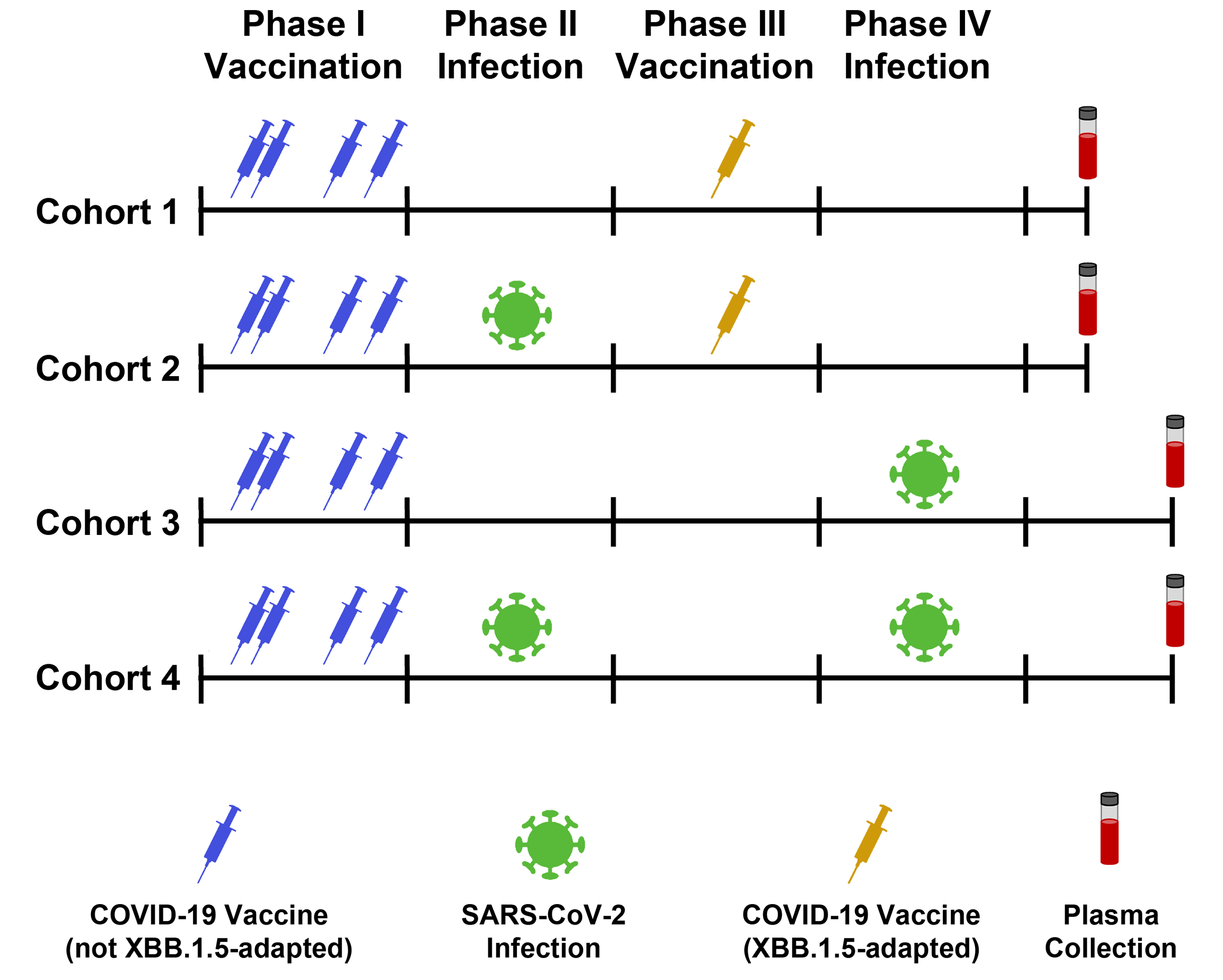

Supplement: Supplementary file 1 [file vaccines-12-00487-s001.zip › SI Figure_4.tif]

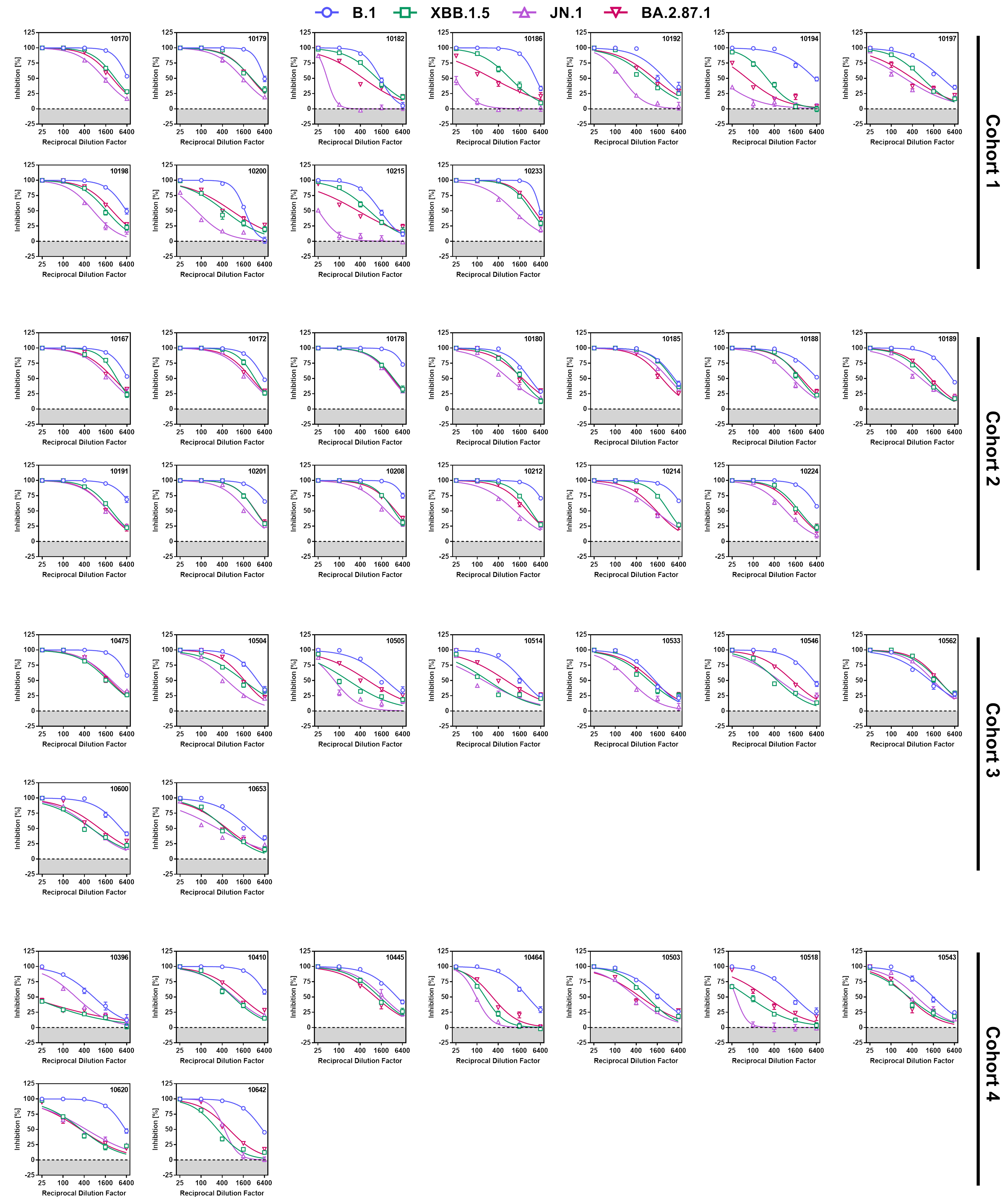

Supplement: Supplementary file 1 [file vaccines-12-00487-s001.zip › SI Figure_5.tif]
